# Cytomegalovirus Retinitis in Primary Immune Deficiency Disease

**DOI:** 10.1155/2018/8125806

**Published:** 2018-09-19

**Authors:** Jia Jeane Ngai, Ka Lung Chong, Shelina Oli Mohamed

**Affiliations:** ^1^Department of Ophthalmology, Hospital Bintulu, Sarawak, Malaysia; ^2^Department of Ophthalmology, Hospital Shah Alam, Selangor, Malaysia

## Abstract

**Introduction:**

To report an unusual case of CMV retinitis in Primary Immune Deficiency Disease (PIDD).

**Case Report:**

13-year-old child with combined T and B cell deficiencies was diagnosed of bilateral zone 1 CMV retinitis. Intravitreal injections were unable to be given in a regular and timely manner under general anaesthesia due to her underlying systemic disease. The child was treated with intravenous ganciclovir for 8 weeks until eventual resolution of the retinitis. However, visual acuity deteriorated due to progressive optic nerve involvement.

**Conclusion:**

Paediatric patients often do not notice subtle symptoms of CMV retinitis. Although ocular manifestations are uncommon in PIDD, recognition and high index of suspicion will allow for timely referral, diagnosis, and treatment to be instituted for better visual outcomes.

## 1. Introduction

Primary Immune Deficiency Disease (PIDD) is a group of genetic disorders that affect the innate and adaptive immune system [1], causing recurrent chronic infections. Common ocular manifestations in combined immunodeficiency include CMV retinitis followed by chorioretinitis and optic neuritis [2]. Cytomegalovirus (CMV) retinitis is a sight threatening condition which is more frequently reported in children with acquired immunodeficiency syndrome compared to other immunosuppressed condition such as post bone marrow or renal transplantation, PIDD, or chemotherapy for leukaemia. As such, present treatment recommendations focus mainly on CMV retinitis in HIV-infected children [3]. Unfortunately, optimal treatment for CMV retinitis in children with PIDD remains unknown. We found limited published reports describing CMV retinitis in PIDD [2, 4, 5]. Here, we present our case findings and treatment approach of CMV retinitis in a child with combined T and B cell deficiencies.

## 2. Case Report

**Figure 1:**
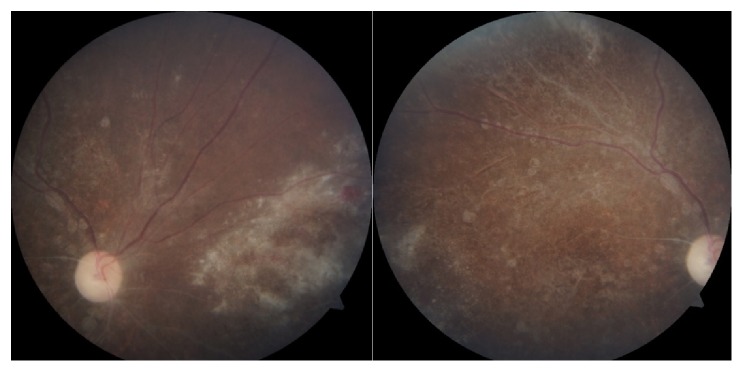
Right eye prior to treatment.

**Figure 2:**
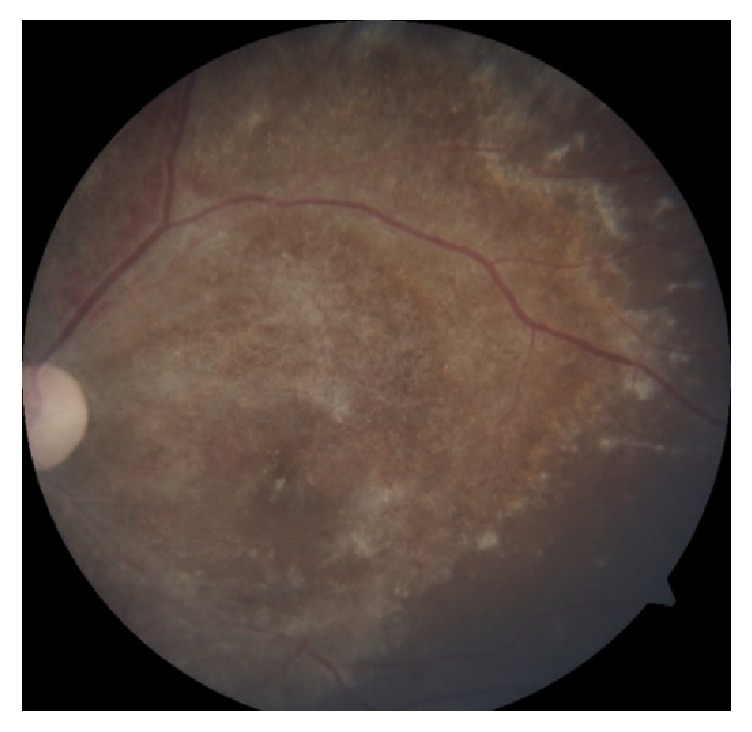
Left eye prior to treatment.

A 13-year-old child with learning disability was referred for ophthalmic assessment as she complained of bilateral blurring of vision. At presentation, her visual acuity was 1/60 in the right eye and 6/18 in the left eye. Relative afferent pupillary defect (RAPD) was present in the right eye. Both eyes anterior segment examination findings were normal. Grade 1 vitritis was noted in the right eye. Funduscopic examination of the right eye revealed a pale optic disc and pigmented scar over the macula with salt and pepper appearance. Intense retinitis with focal areas of haemorrhage was present nasally (Figure 1). There was mild pallor of the left optic disc with macula and peripheral granular retinitis (Figure 2).

Systematically, she was diagnosed with combined T and B cell deficiencies by the immunologist at the age of 11. At that time, she presented with high fever, recurrent episodes of diarrhoea, oral thrush, and failure to thrive, with the weight of only 12kg. PIDD screening showed low T cell, very low B cell counts, and low immunoglobulin levels (Table 1). Her systemic therapy consisted of 3 weekly intravenous immunoglobulin, sulfamethoxazole, and trimethoprim prophylaxis as well as empirical therapy for fungal infection which include syrup fluconazole 6mg/kg/day and syrup nystatin 1ml QID. She was also treated for CMV colitis as HPE of the colon showed CMV inclusion bodies. She completed 6 weeks of intravenous ganciclovir 3 months prior to presentation of her ophthalmic symptoms.

**Figure 3:**
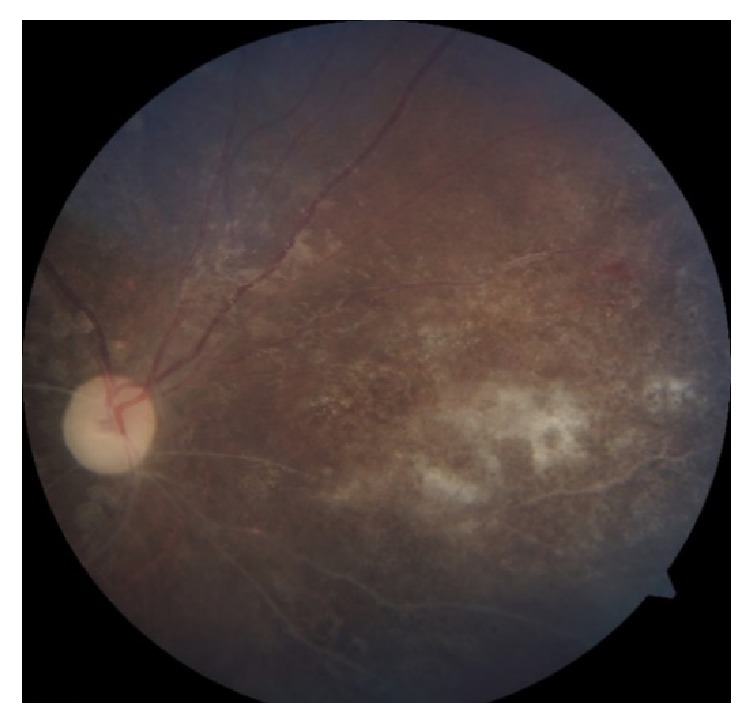
Right eye 2 weeks after IV ganciclovir.

**Figure 4:**
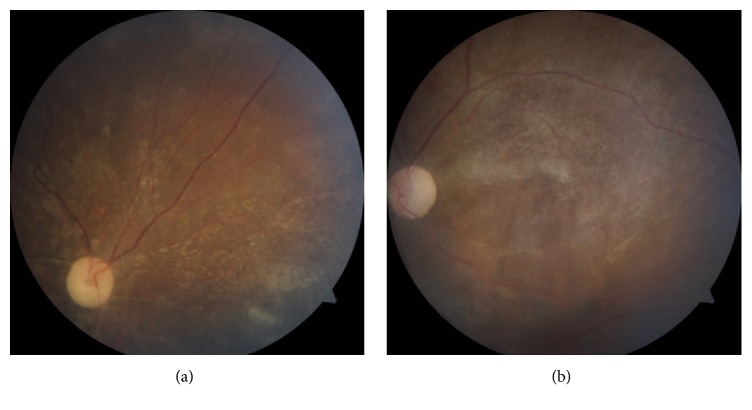
Right eye after completion of 8 weeks of IV ganciclovir (a). Left eye after completion of 8 weeks of IV ganciclovir (b).

We diagnosed the child of having bilateral eye CMV retinitis based on typical fundus features and history of treated CMV colitis. She was planned for right eye intravitreal ganciclovir injection in view of poor visual function with posterior pole involvement. However, she was deemed unfit to undergo general anaesthesia due to concomitant hospital acquired pneumonia. IV ganciclovir 75mg (6mg/kg) 12 hourly was started and good response was noted after 2 weeks of therapy (Figure 3). The treatment was continued for 8 weeks until the retinitis lesions had healed with scarring (Figure 4). However, the right visual acuity reduced to light perception and improved to 6/9 in the left eye.

## 3. Discussion

CMV retinitis can occur in patients with impaired T- cell response such as in solid organ transplant, bone marrow transplant, PIDD, AIDS, or those on immunosuppressive therapy [6]. Although anti-CMV antibody is produced initially as a result of CMV infection, CD4+ T cells and CD8+ T cells play the most important role [6]. The classical fundus appearance of CMV retinitis varies in early and late presentations. In early presentation, it occurs in the peripheral retina with less intense white retinitis, which may not have focal areas of retinal haemorrhages. As the disease progresses, dense white areas of retina necrosis involving the posterior pole, spreading along the vascular arcades, may be seen [7]. The diagnosis of CMV retinitis is mainly clinical, based on classical fundus findings. It can be further confirmed with aqueous CMV polymerase chain reaction (PCR) analysis [6].

The diagnosis of CMV retinitis in this child was established late as evident by bilateral and zone 1 involvement. Patients, especially children with early peripheral CMV retinitis often do not notice the subtle symptoms such as mild blurring of vision, loss of peripheral vision, floaters, or scotoma. Significant visual loss in her right eye is mainly attributed to aggressive extension of retinitis to the optic nerve and macula. Despite achieving resolution of retinitis with antiviral therapy, her visual outcome remained poor.

In AIDS patients, extraocular CMV disease seems to be a strong predisposing factor for developing CMV retinitis. A study has shown that 85% AIDS patients with extraocular CMV disease subsequently developed CMV retinitis after a mean of 6.4 months [8]. The child was diagnosed with CMV colitis and was treated with 6 weeks of IV ganciclovir. She never had any ophthalmic evaluation prior to this. This posed a diagnostic dilemma on whether the initial presentation was due to partial treatment, treatment failure, or relapse of CMV retinitis. In these circumstances, we recommend that such patient should be referred for ophthalmic evaluation before commencement of treatment for extraocular CMV disease.

Treatment strategies which include choice of antiviral and its duration for CMV retinitis in human immunodeficiency virus (HIV) negative patients are vague, particularly in the paediatric group. We have extrapolated the treatment approach used in HIV-infected children with CMV disease in this case. IV ganciclovir, IV foscarnet, IV cidofovir and oral valganciclovir are choices of antiviral available for CMV retinitis in both adults and children. Ganciclovir sustained-release intraocular implant is no longer being manufactured. The treatment guidelines from the Centers for Disease Control and Prevention, the National Institutes of Health, and the HIV Medicine Association of the Infectious Diseases Society of America recommended IV ganciclovir 6 mg/kg body weight/dose administered 12 hourly as initial treatment for HIV-infected infants with CMV disease [3].

Valganciclovir, the oral prodrug of ganciclovir, has been well established to be as effective as IV ganciclovir for induction and maintenance therapy of CMV retinitis in HIV adult patients [9]. Data for the usage of oral valganciclovir for initial treatment in CMV retinitis in children is still lacking. However, oral valganciclovir can be considered for chronic maintenance therapy [3]. Recommended dosage for valganciclovir in paediatric patients (one daily milligram dose) is 7 x body surface area x creatinine clearance (calculated using a modified Schwartz formula). If the calculated Schwartz creatinine clearance exceeds 150 mL/min/1.73m2, then a maximum value of 150 mL/min/1.73m2 should be used [10].

For Zone 1 disease, intensive administration of intravitreal ganciclovir and/or foscarnet given concomitantly with systemic antiviral has been suggested [11]. Repeated intravitreal ganciclovir results in high intraocular antiviral levels could significantly reduce the amount of CMV in CMV retinitis [12]. However, intraocular therapy when administered alone, does not give protection to the fellow eye or additional cytomegalovirus disease, and thus should be combined with systemic antiviral [13]. Meanwhile, small peripheral lesions usually resolve with systemic therapy alone without local treatment [3]. As the usage of cidofovir has not been well established in children, it is considered only if other antivirals failed. Combination therapy of ganciclovir with foscarnet, although has been shown to delay progression of retinitis, should be weighed carefully against its potential risk of nephrotoxicity and neurotoxicity [3].

The severity and location of retinitis, patient's systemic condition, and complications and response to treatment need to be tailored accordingly. IV ganciclovir 6 mg/kg body weight/dose administered 12 hourly was started in this case. We intended for intravitreal injection in this case but, after discussion with the paediatricians and anaesthetists, biweekly intravitreal injection was not feasible due to her multiple systemic comorbidities. Within 2 weeks of treatment with IV ganciclovir, we noted substantial regression of retinitis in the child. Hence, additional antiviral agents were not considered. After complete regression of retinitis with IV ganciclovir, oral valganciclovir as a maintenance is preferred due to its more convenient administration. Unfortunately, it was not available in our setting. As all current antiviral therapies for CMV retinitis are virostatic [14], the definitive treatment in her condition with underlying primary immunodeficiency disease would be bone marrow transplantation with a related matched donor [15]. Unless immune recovery ensues, she will require lifelong monitoring and antiviral therapy for prevention of CMV reactivation.

## 4. Conclusion

In this case, zone 1 CMV retinitis was diagnosed late and suboptimal treatment was given due to her underlying systemic disease. Hence, although there was eventual resolution of the retinitis after 8 weeks of intravenous therapy, the visual acuity deteriorated due to progressive optic nerve involvement from the retinitis. This highlights the importance of a high index of suspicion with timely referral to prevent irreversible visual loss in these patients. The importance of good teamwork between the Paediatrician, Anaesthetist, and Ophthalmologist may ensure optimal treatment, thus preventing blinding complications of CMV retinitis in children with PIDD.

## Figures and Tables

**(a):** 

**Lymphocytes Subset**			**Normal range for age 7-17**	
	%	**[x10 (6)L]**	%	**[x10 (6)L]**
Total T cells	38	802	66-76	1400-2000
Total B cells	0	7	12-22	300-500
Th cells (CD4)	10	176	33-41	700-1000
Ts cells (CD8)	24	440	27-35	600-900
NK cells	59	1428	9-16	200-600

**(b):** 

**Immunoglobulins**	**Result**	**Reference range**
IgG	613	931-1916
IgA	134	70-473
IgM	117	34-265
IgE	121	<165
